# 3-[(*E*)-Benzyl­idene]indolin-2-one

**DOI:** 10.1107/S1600536812024762

**Published:** 2012-06-13

**Authors:** Abdullah M. Asiri, Mohie E. M. Zayed, Seik Weng Ng, Edward R. T. Tiekink

**Affiliations:** a Chemistry Department, Faculty of Science, King Abdulaziz University, PO Box 80203, Jeddah, Saudi Arabia; b Department of Chemistry, University of Malaya, 50603 Kuala Lumpur, Malaysia

## Abstract

In the title indolin-2-one derivative, C_15_H_11_NO, the phenyl ring and the oxoindoline fused-ring system (r.m.s. deviation = 0.011 Å) are aligned at 48.52 (6)°. In the crystal, inversion-related mol­ecules form an N—H⋯O hydrogen-bonded dimer *via* an eight-membered {⋯HNCO}_2_ synthon. The dimeric aggregates are linked into a three-dimensional architecture *via* C—H⋯O and π–π inter­actions between the five- and six-membered rings of the fused ring system, with an inter-centroid distance of 3.4538 (8) Å.

## Related literature

For the structure of the *Z*-isomer of the title compound, see: Milanesio *et al.* (2000 ▶). For background to related thia­zoles, see: Badahdaha *et al.* (2009 ▶).
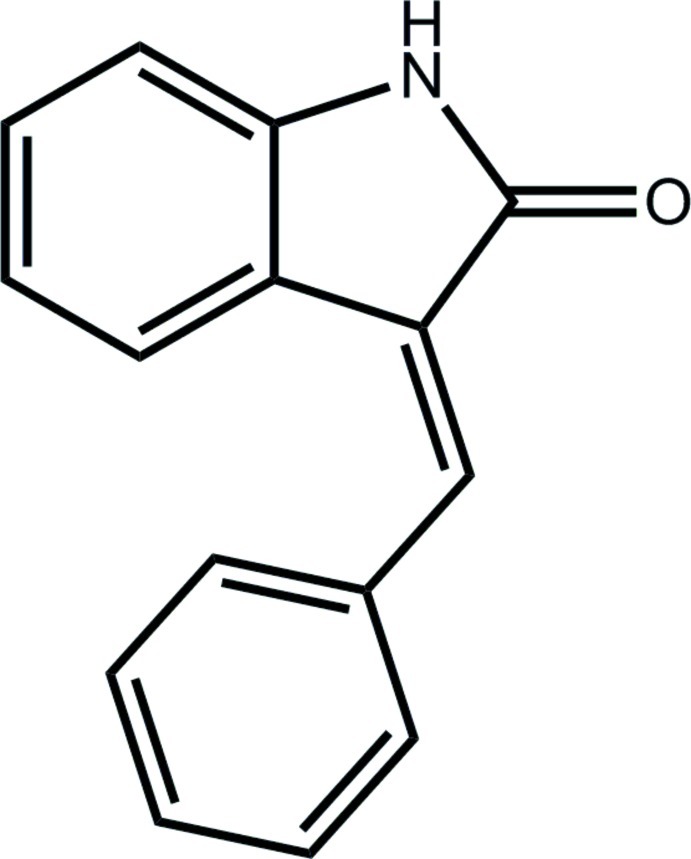


## Experimental

### 

#### Crystal data

C_15_H_11_NO
*M* *_r_* = 221.25
Monoclinic, 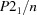

*a* = 3.9796 (3) Å
*b* = 22.2266 (19) Å
*c* = 12.2484 (10) Å
β = 95.027 (1)°
*V* = 1079.24 (15) Å^3^
*Z* = 4
Mo *K*α radiation
μ = 0.09 mm^−1^
*T* = 100 K
0.30 × 0.20 × 0.10 mm

#### Data collection

Bruker SMART APEX diffractometer
6831 measured reflections
2460 independent reflections
2153 reflections with *I* > 2σ(*I*)
*R*_int_ = 0.023

#### Refinement

*R*[*F* ^2^ > 2σ(*F* ^2^)] = 0.038
*wR*(*F* ^2^) = 0.107
*S* = 1.03
2460 reflections
158 parameters
1 restraint
H atoms treated by a mixture of independent and constrained refinement
Δρ_max_ = 0.27 e Å^−3^
Δρ_min_ = −0.26 e Å^−3^

### 

Data collection: *APEX2* (Bruker, 2009 ▶); cell refinement: *SAINT* (Bruker, 2009 ▶); data reduction: *SAINT*; program(s) used to solve structure: *SHELXS97* (Sheldrick, 2008 ▶); program(s) used to refine structure: *SHELXL97* (Sheldrick, 2008 ▶); molecular graphics: *ORTEP-3* (Farrugia, 1997 ▶) and *DIAMOND* (Brandenburg, 2006 ▶); software used to prepare material for publication: *publCIF* (Westrip, 2010 ▶).

## Supplementary Material

Crystal structure: contains datablock(s) global, I. DOI: 10.1107/S1600536812024762/mw2072sup1.cif

Structure factors: contains datablock(s) I. DOI: 10.1107/S1600536812024762/mw2072Isup2.hkl

Supplementary material file. DOI: 10.1107/S1600536812024762/mw2072Isup3.cml

Additional supplementary materials:  crystallographic information; 3D view; checkCIF report

## Figures and Tables

**Table 1:** Hydrogen-bond geometry (Å, °)

*D*—H⋯*A*	*D*—H	H⋯*A*	*D*⋯*A*	*D*—H⋯*A*
N1—H1N⋯O1^i^	0.87 (1)	1.98 (1)	2.846 (1)	172 (2)
C13—H13⋯O1^ii^	0.95	2.57	3.2535 (15)	129

Symmetry codes: (i) 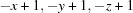; (ii) 
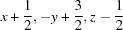
.

**Fig. 1.**
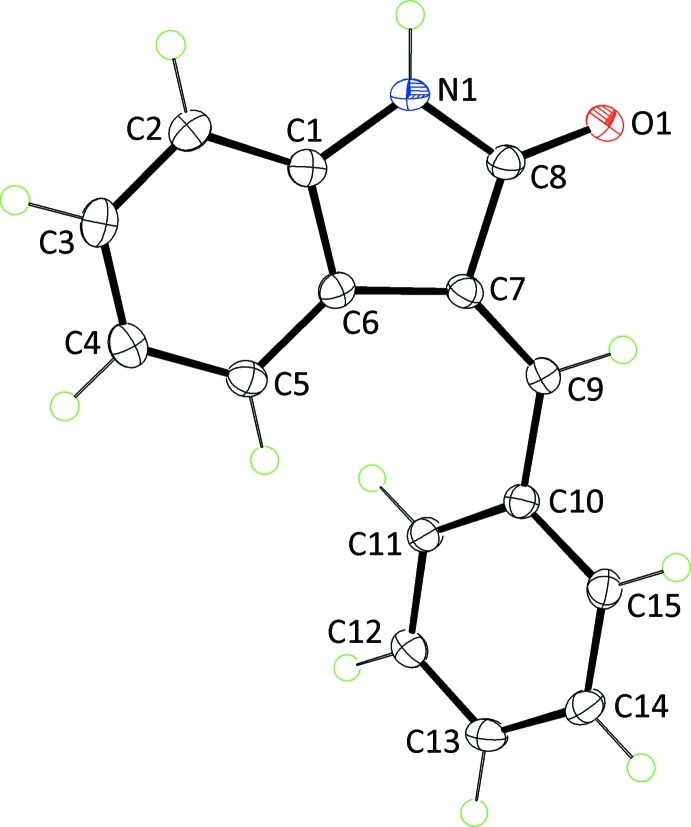
The molecular structure of (I) showing the atom-labelling scheme and displacement ellipsoids at the 50% probability level.

**Fig. 2.**
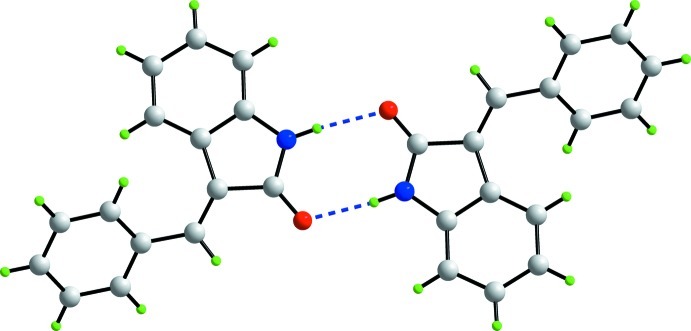
A view of the centrosymmetric dimer in (I) mediated by N—H···O hydrogen bonds shown as blue dashed lines.

**Fig. 3.**
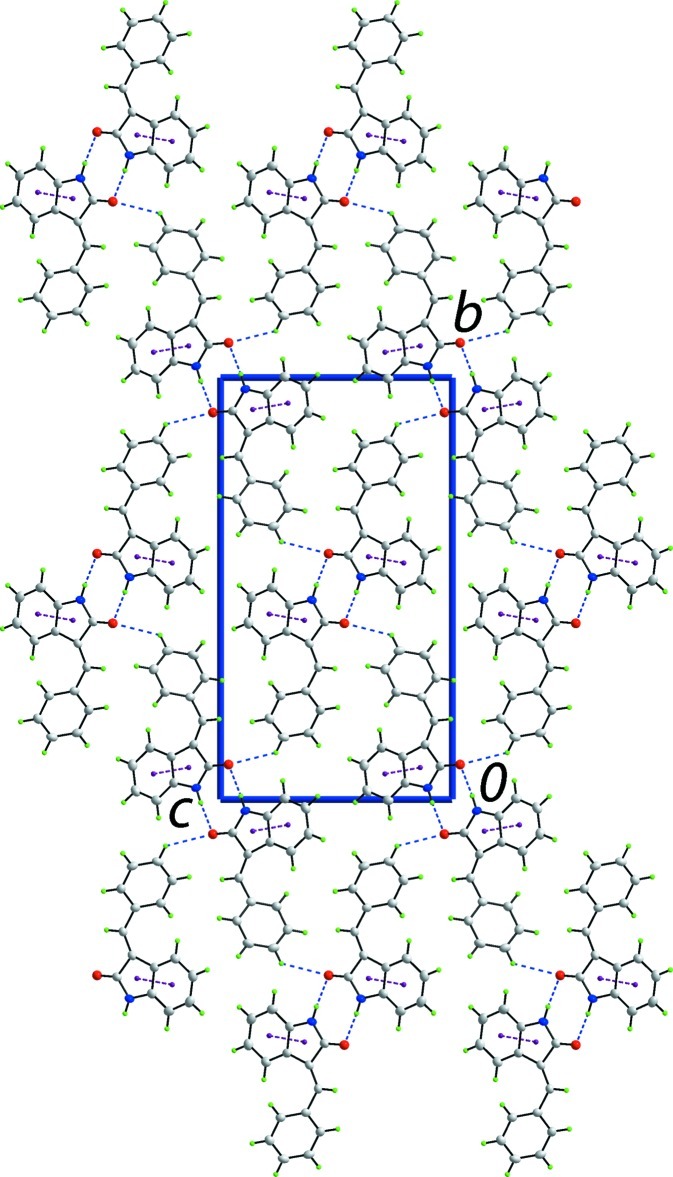
A view in projection down the a axis of the unit-cell contents of (I), the direction of the stacking of supramolecular layers. The N—H···O, C—H···O and π—π interactions are shown as blue, orange and purple dashed lines, respectively.

## supplementary crystallographic information

### Comment

The title compound (I) was isolated as a side-product in the attempted synthesis of thiazole derivatives (Badahdaha *et al.*, 2009) of interest owing to putative biological activity. The indolin-2-onyl fused-ring in (I), Fig. 1, is planar with the r.m.s. deviation being 0.011 Å; the O1 and C9 atoms lie -0.016 (1) and 0.089 (1) Å out of the plane, respectively. The phenyl group is twisted out of this plane, forming a dihedral angle of 48.52 (6)°. The conformation about the C7═C9 bond [1.3439 (16) Å] is *E*; the structure of the *Z*-isomer is known (Milanesio *et al.*, 2000).

In the crystal packing, inversion related molecules are connected into a dimer *via* N—H···O hydrogen bonds, Fig. 2 and Table 1. Molecules are linked into undulating layers in the *bc* plane *via* C—H···O interactions, Table 1, and are linked along the *a* axis *via*π–π interactions occurring between the five- and six-membered rings of the indolin-2-onyl fused ring system [ring centroid(N1,C1, C6–C7)···centroid(C1–C6)^i^ = 3.4538 (8) Å, angle of inclination = 1.15 (6)° for i: -1 + *x*, *y*, *z*], Fig. 3.

### Experimental

The title compound, an unexpected product, was isolated as a side-product from a reaction between 2-(4-nitrobenzylidene)hydrazinecarbothioamide, triethylamine and *N*-phenyl-2-oxopropanehydrazonoyl chloride in ethanol solution; m.p. 512 K.

### Refinement

Carbon-bound H atoms were placed in calculated positions [C—H = 0.95 Å, *U*_iso_(H) = 1.2*U*_eq_(C)] and were included in the refinement in the riding model approximation. The N-bound H atom was located in a difference Fourier map and was refined with a distance restraint of N—H = 0.86 (1) Å; the *U*_iso_ value was refined.

### Crystal data

**Table:**

C_15_H_11_NO	*F*(000) = 464
*M**_r_* = 221.25	*D*_x_ = 1.362 Mg m^−^^3^
Monoclinic, *P*2_1_/*n*	Mo *K*α radiation, λ = 0.71073 Å
Hall symbol: -P 2yn	Cell parameters from 3166 reflections
*a* = 3.9796 (3) Å	θ = 2.5–28.3°
*b* = 22.2266 (19) Å	µ = 0.09 mm^−^^1^
*c* = 12.2484 (10) Å	*T* = 100 K
β = 95.027 (1)°	Prism, orange
*V* = 1079.24 (15) Å^3^	0.30 × 0.20 × 0.10 mm
*Z* = 4	

### Data collection

**Table:**

Bruker SMART APEX diffractometer	2153 reflections with *I* > 2σ(*I*)
Radiation source: fine-focus sealed tube	*R*_int_ = 0.023
Graphite monochromator	θ_max_ = 27.5°, θ_min_ = 1.8°
ω scans	*h* = −3→5
6831 measured reflections	*k* = −28→28
2460 independent reflections	*l* = −15→15

### Refinement

**Table:**

Refinement on *F*^2^	Primary atom site location: structure-invariant direct methods
Least-squares matrix: full	Secondary atom site location: difference Fourier map
*R*[*F*^2^ > 2σ(*F*^2^)] = 0.038	Hydrogen site location: inferred from neighbouring sites
*wR*(*F*^2^) = 0.107	H atoms treated by a mixture of independent and constrained refinement
*S* = 1.03	*w* = 1/[σ^2^(*F*_o_^2^) + (0.0633*P*)^2^ + 0.2809*P*] where *P* = (*F*_o_^2^ + 2*F*_c_^2^)/3
2460 reflections	(Δ/σ)_max_ = 0.001
158 parameters	Δρ_max_ = 0.27 e Å^−^^3^
1 restraint	Δρ_min_ = −0.26 e Å^−^^3^

### Fractional atomic coordinates and isotropic or equivalent isotropic displacement parameters (Å^2^)

**Table:**

	*x*	*y*	*z*	*U*_iso_*/*U*_eq_	
O1	0.5372 (2)	0.58258 (4)	0.53379 (7)	0.0236 (2)	
N1	0.7366 (3)	0.52725 (4)	0.39388 (8)	0.0183 (2)	
H1N	0.668 (4)	0.4920 (5)	0.4133 (12)	0.032 (4)*	
C1	0.8893 (3)	0.53924 (5)	0.29780 (9)	0.0167 (2)	
C2	0.9905 (3)	0.49792 (5)	0.22291 (10)	0.0194 (3)	
H2	0.9556	0.4560	0.2321	0.023*	
C3	1.1456 (3)	0.52014 (6)	0.13341 (10)	0.0211 (3)	
H3	1.2194	0.4929	0.0808	0.025*	
C4	1.1938 (3)	0.58149 (6)	0.11990 (10)	0.0207 (3)	
H4	1.2987	0.5956	0.0580	0.025*	
C5	1.0897 (3)	0.62256 (5)	0.19619 (9)	0.0184 (2)	
H5	1.1241	0.6645	0.1866	0.022*	
C6	0.9350 (3)	0.60162 (5)	0.28646 (9)	0.0159 (2)	
C7	0.8052 (3)	0.62991 (5)	0.38267 (9)	0.0167 (2)	
C8	0.6761 (3)	0.57888 (5)	0.44818 (9)	0.0180 (3)	
C9	0.7925 (3)	0.68631 (5)	0.42166 (9)	0.0176 (2)	
H9	0.7175	0.6901	0.4929	0.021*	
C10	0.8793 (3)	0.74284 (5)	0.36866 (9)	0.0167 (2)	
C11	0.7759 (3)	0.75418 (5)	0.25856 (9)	0.0179 (2)	
H11	0.6540	0.7243	0.2161	0.021*	
C12	0.8496 (3)	0.80862 (5)	0.21070 (10)	0.0194 (3)	
H12	0.7756	0.8160	0.1360	0.023*	
C13	1.0311 (3)	0.85235 (5)	0.27155 (10)	0.0199 (3)	
H13	1.0862	0.8892	0.2382	0.024*	
C14	1.1317 (3)	0.84202 (5)	0.38124 (10)	0.0203 (3)	
H14	1.2557	0.8719	0.4231	0.024*	
C15	1.0516 (3)	0.78813 (5)	0.43000 (9)	0.0191 (3)	
H15	1.1145	0.7819	0.5058	0.023*	

### Atomic displacement parameters (Å^2^)

**Table:**

	*U*^11^	*U*^22^	*U*^33^	*U*^12^	*U*^13^	*U*^23^
O1	0.0347 (5)	0.0183 (4)	0.0189 (4)	−0.0028 (4)	0.0088 (4)	0.0011 (3)
N1	0.0234 (5)	0.0130 (5)	0.0186 (5)	−0.0015 (4)	0.0024 (4)	0.0020 (4)
C1	0.0150 (5)	0.0178 (6)	0.0167 (5)	0.0001 (4)	−0.0015 (4)	0.0013 (4)
C2	0.0178 (5)	0.0177 (6)	0.0222 (6)	0.0018 (4)	−0.0010 (4)	−0.0010 (4)
C3	0.0191 (5)	0.0246 (6)	0.0193 (6)	0.0039 (5)	0.0001 (4)	−0.0044 (5)
C4	0.0179 (5)	0.0265 (6)	0.0179 (6)	0.0008 (5)	0.0019 (4)	0.0008 (5)
C5	0.0164 (5)	0.0190 (6)	0.0196 (6)	−0.0018 (4)	0.0008 (4)	0.0016 (4)
C6	0.0146 (5)	0.0163 (5)	0.0163 (5)	−0.0004 (4)	−0.0017 (4)	−0.0002 (4)
C7	0.0177 (5)	0.0170 (6)	0.0153 (5)	−0.0017 (4)	0.0004 (4)	0.0025 (4)
C8	0.0209 (6)	0.0157 (6)	0.0171 (5)	−0.0015 (4)	−0.0006 (4)	0.0016 (4)
C9	0.0200 (5)	0.0180 (6)	0.0148 (5)	−0.0005 (4)	0.0020 (4)	0.0006 (4)
C10	0.0172 (5)	0.0146 (5)	0.0188 (5)	0.0008 (4)	0.0046 (4)	−0.0005 (4)
C11	0.0183 (5)	0.0164 (5)	0.0189 (6)	−0.0009 (4)	0.0018 (4)	−0.0009 (4)
C12	0.0200 (6)	0.0202 (6)	0.0181 (5)	0.0015 (4)	0.0032 (4)	0.0022 (4)
C13	0.0196 (6)	0.0151 (5)	0.0255 (6)	−0.0002 (4)	0.0058 (4)	0.0028 (4)
C14	0.0214 (6)	0.0152 (5)	0.0244 (6)	−0.0020 (4)	0.0025 (4)	−0.0038 (4)
C15	0.0224 (6)	0.0182 (6)	0.0167 (5)	0.0015 (4)	0.0018 (4)	−0.0013 (4)

### Geometric parameters (Å, º)

**Table:**

O1—C8	1.2302 (14)	C7—C9	1.3439 (16)
N1—C8	1.3585 (15)	C7—C8	1.5054 (15)
N1—C1	1.3968 (14)	C9—C10	1.4693 (15)
N1—H1N	0.87 (1)	C9—H9	0.9500
C1—C2	1.3825 (16)	C10—C11	1.3978 (16)
C1—C6	1.4068 (16)	C10—C15	1.3993 (16)
C2—C3	1.3946 (17)	C11—C12	1.3871 (16)
C2—H2	0.9500	C11—H11	0.9500
C3—C4	1.3890 (18)	C12—C13	1.3884 (17)
C3—H3	0.9500	C12—H12	0.9500
C4—C5	1.3950 (16)	C13—C14	1.3871 (17)
C4—H4	0.9500	C13—H13	0.9500
C5—C6	1.3919 (15)	C14—C15	1.3881 (16)
C5—H5	0.9500	C14—H14	0.9500
C6—C7	1.4689 (15)	C15—H15	0.9500
			
C8—N1—C1	111.10 (9)	O1—C8—N1	125.86 (10)
C8—N1—H1N	123.7 (11)	O1—C8—C7	127.13 (10)
C1—N1—H1N	124.9 (11)	N1—C8—C7	106.99 (10)
C2—C1—N1	127.28 (11)	C7—C9—C10	128.54 (10)
C2—C1—C6	122.79 (11)	C7—C9—H9	115.7
N1—C1—C6	109.93 (10)	C10—C9—H9	115.7
C1—C2—C3	117.44 (11)	C11—C10—C15	118.50 (10)
C1—C2—H2	121.3	C11—C10—C9	121.32 (10)
C3—C2—H2	121.3	C15—C10—C9	120.08 (10)
C4—C3—C2	121.10 (11)	C12—C11—C10	120.65 (11)
C4—C3—H3	119.4	C12—C11—H11	119.7
C2—C3—H3	119.4	C10—C11—H11	119.7
C3—C4—C5	120.69 (11)	C13—C12—C11	120.20 (11)
C3—C4—H4	119.7	C13—C12—H12	119.9
C5—C4—H4	119.7	C11—C12—H12	119.9
C4—C5—C6	119.42 (11)	C14—C13—C12	119.78 (11)
C4—C5—H5	120.3	C14—C13—H13	120.1
C6—C5—H5	120.3	C12—C13—H13	120.1
C5—C6—C1	118.55 (10)	C13—C14—C15	120.12 (11)
C5—C6—C7	134.85 (11)	C13—C14—H14	119.9
C1—C6—C7	106.56 (10)	C15—C14—H14	119.9
C9—C7—C6	135.33 (11)	C14—C15—C10	120.67 (11)
C9—C7—C8	119.22 (10)	C14—C15—H15	119.7
C6—C7—C8	105.40 (9)	C10—C15—H15	119.7
			
C8—N1—C1—C2	179.20 (11)	C1—N1—C8—C7	−0.80 (13)
C8—N1—C1—C6	0.09 (13)	C9—C7—C8—O1	4.54 (18)
N1—C1—C2—C3	−178.59 (11)	C6—C7—C8—O1	−177.79 (11)
C6—C1—C2—C3	0.41 (16)	C9—C7—C8—N1	−176.49 (10)
C1—C2—C3—C4	−0.48 (17)	C6—C7—C8—N1	1.18 (12)
C2—C3—C4—C5	0.40 (17)	C6—C7—C9—C10	8.2 (2)
C3—C4—C5—C6	−0.23 (17)	C8—C7—C9—C10	−175.00 (11)
C4—C5—C6—C1	0.15 (16)	C7—C9—C10—C11	43.93 (18)
C4—C5—C6—C7	177.75 (11)	C7—C9—C10—C15	−139.89 (13)
C2—C1—C6—C5	−0.25 (16)	C15—C10—C11—C12	1.47 (17)
N1—C1—C6—C5	178.91 (10)	C9—C10—C11—C12	177.71 (10)
C2—C1—C6—C7	−178.47 (10)	C10—C11—C12—C13	0.77 (17)
N1—C1—C6—C7	0.68 (12)	C11—C12—C13—C14	−1.57 (17)
C5—C6—C7—C9	−1.8 (2)	C12—C13—C14—C15	0.09 (18)
C1—C6—C7—C9	175.99 (13)	C13—C14—C15—C10	2.20 (18)
C5—C6—C7—C8	−178.91 (12)	C11—C10—C15—C14	−2.95 (17)
C1—C6—C7—C8	−1.11 (11)	C9—C10—C15—C14	−179.24 (10)
C1—N1—C8—O1	178.19 (11)		

### Hydrogen-bond geometry (Å, º)

**Table:**

*D*—H···*A*	*D*—H	H···*A*	*D*···*A*	*D*—H···*A*
N1—H1*N*···O1^i^	0.87 (1)	1.98 (1)	2.846 (1)	172 (2)
C13—H13···O1^ii^	0.95	2.57	3.2535 (15)	129

Symmetry codes: (i) −*x*+1, −*y*+1, −*z*+1; (ii) *x*+1/2, −*y*+3/2, *z*−1/2.
